# The New England 4G framework for the treatment of a common health concerns: a gambling case analysis

**DOI:** 10.1186/s40405-016-0019-7

**Published:** 2016-10-24

**Authors:** Barry Tolchard, Cynthia M. Stuhlmiller

**Affiliations:** 1 School of Nursing, University at Buffalo, Buffalo, NY 14212 USA; 2 School of Health, University of New England, Armidale, NSW 2351 Australia

**Keywords:** Problem gambling, Rural health, Self-help, Cognitive behavioral therapy

## Abstract

Approaches using self-help have proved successful at treating a range of mental and physical conditions. Guidance by a trained worker enhances the effects of self-help materials, in particular those based on Cognitive-Behavior Therapy. In the United Kingdom, the Improving Access to Psychological Therapies (IAPT) program was introduced to provide better outcomes for people experiencing mild or moderate anxiety and depression. This stepped care approach included low intensity, guided self-help offered by a newly trained workforce of Psychological Wellbeing Workers. The IAPT program has been extensively evaluated and shown to be cost effective and leads to positive treatment outcomes. This paper describes how the IAPT model has been adapted for use in Australia with gamblers. Two case studies illustrate the application of this guided approach to systematically accessing existing self-help treatments for problem gamblers. Assessment information is gathered, before a plan of action, including a problem statement and achievable goals, is agreed upon by the worker and the person with the gambling problem. The worker then gives the person options based on self-help CBT interventions and, once an option has been chosen, the worker guides the person as they work through various activities. The benefits of this approach are discussed.

## Introduction

It is recognized that early treatment for people with mental and physical conditions prevents a range of concomitant issues, including co-morbid mental health problems and deteriorating physical health (Jorm 2012). Cognitive behaviorally based therapies (CBT) are considered the most efficacious of the non-pharmacological treatments (Stuhlmiller and Tolchard 2009). However, the demand for CBT has long outstripped the available qualified health practitioners able to provide it. In response to this disparity, two solutions have emerged to refine and streamline therapy to ensure that: (1) only the active ingredients necessary for outcomes are delivered; and (2) greater use of self-help is encouraged and facilitated (Norman and Ryrie 2013). The extensive work of Marks and colleagues (Marks 2009; Marks et al. 1977) was based on these solutions. They developed CBT self-help strategies focusing on essential techniques of Cognitive Restructuring, Behavioural Activation, or Graded Exposure (Mataix-Cols and Marks 2006). They also established that the routine aspects of CBT could be delivered as effectively via print-based or internet programs, thus reducing the face-to-face time necessary (Marks et al. 2007; Stuhlmiller and Tolchard 2009). This foundational work informed the UK National Institute of Health and Clinical Excellence (NICE) policy and guidelines for treatment of depression and anxiety (National Institute for Clinical Excellence 2004a, b).

In 2006, it was reported that the cost of depression in the UK neared £7 billion a year (Layard 2006). This triggered an all of government response. A pilot project was established and within a year, positive results had been achieved enabling individuals with anxiety and/or depression to return to work (Clark et al. 2009; Glover et al. 2010; Richards and Suckling 2008, 2009). Based on this success, in 2010 the UK National Health Service mandated that all primary care services provide screening and treatment for anxiety and depression.

A common curriculum and service entitled Improving Access to Psychological Therapies (IAPT) was developed to train low intensity psychological wellbeing practitioners (PWPs) and high intensity therapists in CBT, and to ensure high standards would be established for the national roll-out. IAPT programs have prepared both low and high intensity workers in programs of six months and twelve months duration respectively (Clark 2011). As of 2015, there were eighteen accredited low intensity and nineteen high intensive IAPT training programs offered through UK universities at either the undergraduate or postgraduate level. Three thousand six hundred trainees will have graduated out of a desired target of six thousand by 2016 (Improving Access to Psychological Therapies 2016). Low intensity PWPs can be graduates or undergraduates, whereas high intensity therapists are graduates from many different clinical backgrounds including clinical psychologists, counsellors, nurses, occupational therapists, mental health workers etc.

### CBT-based approach

The CBT approach is largely based on a functional analysis which pulls together the person’s thoughts, feelings, physical responses, and behaviours as they relate to the circumstances before and after the problem experience (Kanter et al. 2005; Sturmey 2011). During treatment, the health worker assists the person to identify these cognitive, physical and behavioural components of situations that represent their main problem. Once uncovered, a number of common strategies are explored with the client. The first is Cognitive Restructuring, which examines errors of thinking and challenges the person to consider alternative realities (Shikatani et al. 2014; Shurick et al. 2012). The second, Behavioural Activation, assists the person by using an activity diary to increase activities related to pleasure and accomplishment and to reduce activities related to a lower sense of achievement (Kanter et al. 2010; Martell et al. 2013). A third, Graded Exposure, aims to support the person in confronting circumstances of discomfort in a repeated and prolonged manner, based on a graded hierarchy of discomfort (Dobson 2009). In addition to these basic treatments, commonly applied techniques assist with co-morbid issues with sleep, anger, or medications and these can be successfully taught to an unskilled workforce. All interventions are supported by self-help materials through which the health worker guides the individual.

### Self-help for problem gambling

Problem gambling was previously considered an impulse control problem until the classification was changed to substance use disorders (Petry 2010). Problem gambling affects between 0.5 and 3.0 % of the population (Becoña 1993; Sassen et al. 2011; Sproston et al. 2012; Wardle 2007; Williams and Volberg 2012). Not only can gambling lead to severe economic and social losses, the overwhelming anxiety and depression experienced with the problem can lead to desperate behaviors such as crime and suicide (Battersby et al. 2006; Park and Stokowski 2011; Thon et al. 2014). A lack of routine screening to assess the level and severity of gambling problems coupled with the associated stigma are the major barriers for problem identification and individuals seeking help.

Self-help materials can overcome these barriers by enabling self-assessment and privacy. Evidence-based tools such as print-based books, manuals, and information or guidelines published on the internet can be readily accessed at no cost and require minimal, if any, clinical involvement (Marks et al. 2007). While only a few studies have evaluated self-help for gambling, the results have been promising, showing improvement of gambling severity and other behavioral indicators over control or wait list conditions (Carlbring and Smit 2008; Hodgins et al. 2001, 2004; LaBrie et al. 2012; Petry 2010; Raylu et al. 2008). Typical approaches to self-help include, online chat rooms, gambling specific websites and online counselling as well as more traditional self-help books with or without therapist contact (Carlbring and Smit 2008; Ladouceur et al. 2015; Raylu et al. 2008).

In a comprehensive review it was found that minimally invasive self-directed resources can assist in remediating gambling related problems among gamblers who do not engage in formal treatment. This review identified self-help based on changing thoughts, improving and promoting activities other than gambling and self-monitoring to be the most effective strategies (Lubman et al. 2015). Further, this study noted that promoting universal strategies could lead to poor take-up by gamblers and may largely be ineffective and suggested individually targeted approaches were more likely to be effective.

### The New England 4G framework as applied to problem gambling

The authors propose that IAPT interventions should be expanded to include specific conditions such as problem gambling, since most health conditions respond favourably to CBT-based help (Hofmann et al. 2012). Furthermore, from their experiences working in regional and rural parts of Australia, the authors wished to address the issues associated with accessing healthcare remotely, resulting in the development of the New England 4G framework.

### Training for New England 4G workers

The framework is based on strict training protocols (Stuhlmiller and Tolchard 2012). Health workers and unqualified health personnel are trained to guide clients in the use of CBT-based self-help materials of various kinds, in particular Cognitive Restructuring, Behavioural Activation, and Graded Exposure techniques. The worker coaches the client to use the online, print-based or other materials that have been chosen to enable self-tailored interventions. Research has shown that in addition to health education, ongoing short focused phone or face-to-fact contact significantly increases the uptake of self-help (Gournay 2006a, b) and this has been implemented in this program. It is documented that the minimum overall time required for health worker input is a 20–30-min assessment with 4–6 follow up phone or face-to-face sessions of 10 min each (Stuhlmiller and Tolchard 2012). However, as described earlier depending on the client, more or less guidance may be needed. These health workers are trained to:

- adopt a collaborative approach to engaging and coaching clients in self-management;
- employ the low intensity method of guiding clients in evidence-based self-help practices;
- help clients to choose the approach that might suit them the best (Cognitive Restructuring or Behavioural Activation or Graded Exposure);
- help clients to generate a succinct health problem statement with cognitive, emotional, behavioural, and autonomic components and to align it with specific, realistic, measurable health goals to address these components;
- teach clients to measure progress based on standardised health measures;
- encourage clients to try a different approach if they are failing to make sufficient progress; and
- increase clients’ health literacy by providing information regarding the prevalence and morbidity of specific client conditions and deliver evidence-base options for help.

The training includes specific evidence-based manuals and workbooks, broadly based on the IAPT manuals but with additional information and a set list of approaches to be taken. Clients work online, over the telephone, or use biblotherapy methods using one approach and are only offered a second option if they are not making sufficient progress.

### Additional training for working with gamblers

While the majority of the IAPT manuals are easily adaptable to work with gamblers, there are specific issues of which workers need to be aware. Cognitive restructuring with gamblers is based on three specific belief constructs related to their (1) understanding of the nature of randomness, (2) overestimation of their chances of winning and (3) the belief in skills influencing chance (Gaboury and Ladouceur 1989). The low intensity use of exposure with gamblers also incorporates a session of guided imaginal exposure, which can be done over the telephone, by using a pre-prepared audio file or by directing the gamblers to online instructions. The exposure needs to be tailored to the gambler’s particular game (slots machines, horses, casinos) and no anti-exposure elements are to be included such as distracting while confronting the urge to gamble or use of mindfulness techniques (Tolchard and Battersby 2013). Finally, behavioural activation would encourage through the use of contingency management (Christensen, 2013).

### The New England framework

**Fig. 1:**
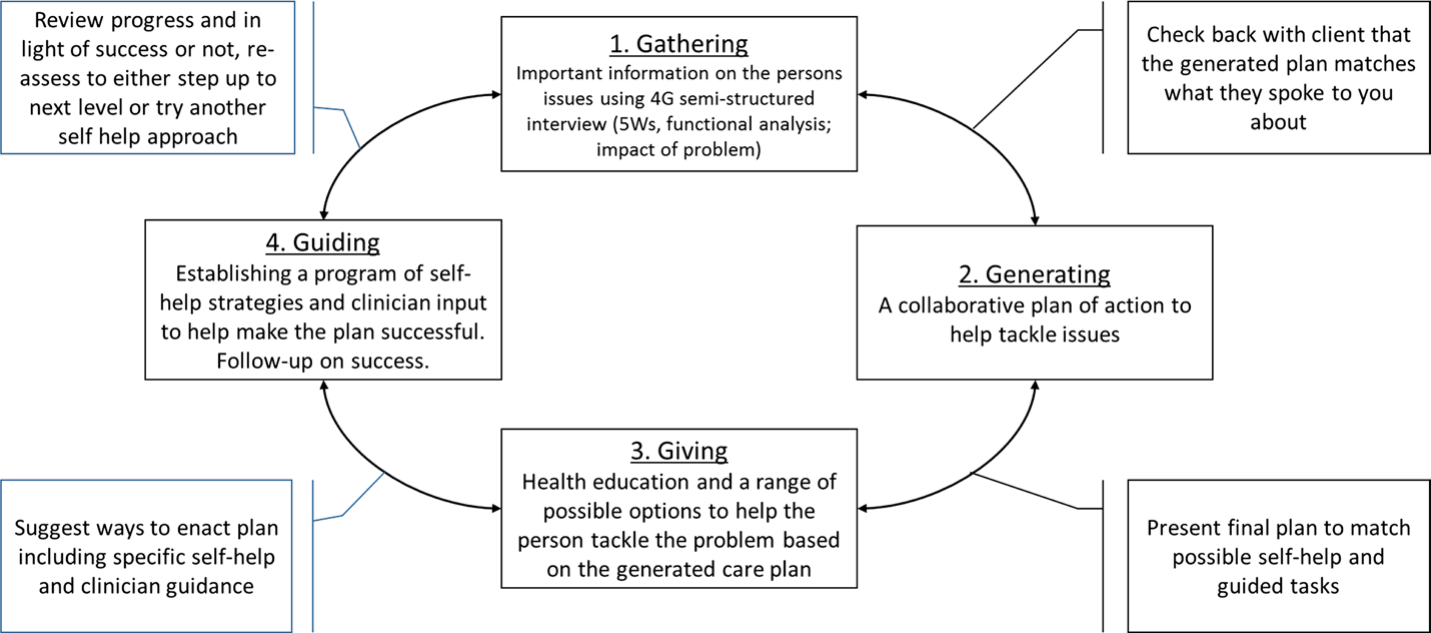
4G process and decisions making stages

The New England 4G framework essentially assists people to access self-help with minimal worker contact. The average amount of time a person spends with their worker averages a little <3 h over 5 sessions of which three sessions are over the telephone (Richards and Suckling 2009; Stuhlmiller and Tolchard 2012). People experiencing problems with their gambling are assessed using the 4G semi-structured interview (Ben-Tovim et al. 2001). Similar to the low intensity interventions in the IAPT program, the elements of the framework include *gathering, giving,* and *guiding* with follow-up. However, the New England 4G framework adds another G to underscore the importance of *generating* a Cognitive Behavioral (CB) plan with a problem statement and relevant goals (Stuhlmiller and Tolchard 2012). To implement this, the worker goes beyond providing health information or coordinating care and helps the person identify a specific health problem, goals, and guides them to use self-health materials. The four elements are described in more detail below (Fig. 1).

In *gathering*, the semi-structured validated assessment is carried out. There are two main elements used to assess the nature of the current problem as the person sees it, not necessarily how others view it. The first uses the 5Ws (what, where, when, with whom and why), which informs a detailed functional analysis of a typical experience using the five aspects or ‘hot-cross bun’ approach (Stuhlmiller and Tolchard 2012). The second element assesses the impact of the problem on all aspects of the person’s life. A series of routine outcomes measure are then completed and repeated throughout the process. The standard measures used are the:

*Patient Health Questionnaire*-*9 (PHQ*-*9)*, a measure of depression which scores each of the nine Diagnostic and Statistical Manual-IV criteria (Kroenke et al. 2010; Manea et al. 2012); *Generalized Anxiety Disorder*-*7 (GAD*-*7)*, a brief measure of state anxiety (Spitzer et al. 2006)*; and Work and Social Adjustment Scale* (WSAS), a measure of the impact of a problem on different aspects of their lives (Mundt et al. 2002). To this any gambling specific measure can be added, such as the Victorian Gambling Screen (VGS) (Tolchard and Battersby 2010) and the Gambling Consumption Screen for Problem Gambling (GCSPG) (Rockloff and Schofield 2004).

The worker is trained to interpret these assessment tools and discuss their interpretation with the client. On completion of the assessment the worker and client then *generates* a plan of action to tackle the problem, which includes a problem statement and relevant goals (Stuhlmiller and Tolchard 2012). This treatment plan is not fixed and provides an initial starting point and is reviewed at every session by the client and worker.

Once this treatment plan is generated, the worker *gives* the client a range of possible approaches to dealing with the identified issues. The client chooses which they believe will be the most likely to assist them with the problems they have identified. Finally, while the client is working on their problem, the worker provides regular support through a range of *guiding* processes. This may include infrequent face-to-face contact, telephone or SMS guidance, or e-mail/internet based support.

## Case descriptions and discussion

The remainder of this article will draw on two case examples to illustrate the application of the New England 4G framework and its outcomes in working with problem gamblers.

The following two cases are typical examples drawn from a larger clinical population from a nurse-led clinic in Adelaide, Australia (Tolchard 2009; Tolchard and Battersby 2001, 2013). They were chosen because they represent different gambling presentations and each gender. The cases will be used to highlight the application of the 4G framework with gamblers. Both clients provided written consent for their health data to be reported. No names have been used to maintain anonymity. Masters prepared nurses with mental health experience provided guided treatment. These nurses were experienced clinicians and so may have influenced the outcome of the guided self-help. However, similar approaches have since been adopted by people with no prior experience in mental health with similar outcomes (Mutamba et al. 2013; van Ginneken et al. 2013).

### Case one: female, electronic gaming machine (EGM) gambler

#### Gathering

The first case analysis involves a divorced woman who has an electronic gaming machine (slot machine) addiction. At the time of the initial assessment she had recently separated from her de facto partner and was living alone in her own home. She was a long-term employee of a large production company working shifts as a team leader.

In the gathering step of the Framework the client was asked to describe her gambling problem using the five w’s. The triggers, cognitive, emotional behavioural, physical features to her experience were gathered and formulated into a ‘hot cross bun’ analysis. She described her experience as follows.

##### Five w’s

*What* My main problem is gambling on EMGs especially when I am stressed or having received money. *Where* I gamble in venues close to family and friends including at the local shopping mall, which is within walking distance from my house. I visit the local casino socially with family and friends for meals and celebrations. However, no one seems to notice that I have a problem controlling my gambling. *When* I do not gamble while at work but I do gamble any other time of day or night. *With whom* While I like to do some socialising, I mostly gamble alone when stressed. *Why* I feel a relief of stress, a sense of escape and entertainment. I am motivated to gamble because I might win.

**Fig. 2:**
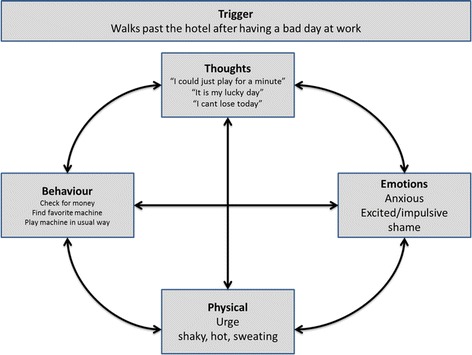
Five aspects of the ‘hot-cross bun’ model for case 1

She described her typical trigger of walking past a hotel after a bad day at work. Her thoughts were “I could just play a minute” “It is my lucky day”, “I can’t lose today”. She was aware of feeling anxious, excited, and impulsive but also some shame. Her physical responses to her thoughts included an urge, shaking, hot, and sweating. Behaviourally, she checked for her money, found her favourite machine, and began gambling. The elements are organised in the “hot cross bun” format as below in Fig. 2.

*Impact* Over the period she had been gambling her losses were approximately $100,000 and at times she gambled her whole fortnightly income in one session. Furthermore, she had taken a second mortgage on her house, pawned items, borrowed heavily from family and friends, and accumulated credit card debt. In addition to her losses she also experienced mood swings when gambling with episodes of depression, anger, and frustration. These were all heightened and increased when she had been losing. Her gambling affected her sleep patterns as she continually had thoughts and images of games, winning, the EGM venues, and the casino. She described poor eating patterns, eating the “wrong foods” but when gambling she often would not eat at all. *Suicidal intent* She had previously planned suicide 15 years earlier with the aim of taking an overdose of medication. However, she did not follow through with this plan. More recently she had again had suicidal ideation, although she had not acted on this. At the time of treatment, she reported no suicidal ideation. *Onset* She first gambled when she escaped from her violent ex-husband, which was about when the casino opened. Her gambling worsened when the EGMs were introduced locally. *Measures* She scored in the moderate range on all measures except the specific gambling screen (PHQ-9 = 15; GAD-7 = 13; WSAS = 3; Victorian Gambling Screen (VGS) = 22 (problem gambler).

#### Generating

The problem statement provides the client and worker a subjective sense of the scale of the problem. This is then graded to allow for change over time to be recorded. The statement is measured using a 9 point Likert type scale where zero = no problem and eight = a very severe problem.

*Problem statement* I experience a strong urge to gamble on the Electronic Gaming Machines (Pokies) close to home, at any time of day when not working and always alone whenever I feel stressed or have money and I think I can just play for a short time or my big win is going to happen (8/8).

The goals are derived from the problem statement and direct the guided self-help. As with the problem statement they are subjective statements that are then measured using the nine point Likert scale. The range is zero = *“I am able to achieve this all of the time”* to eight = *“I am unable to achieve this at all”*

*Goal 1* To be able to enter any venues where there are Pokies and enjoy a meal alone without gambling regardless of whether I feel stressed or not (8/8).

*Goal 2* To carry any sum of money while shopping without going to a venue and gambling (8/8).

#### Giving

This client experienced two distinct issues related to her gambling urge—when she had money, or when she was feeling stressed. To deal with these issues overall, Graded Exposure (GE) with response prevention was explained to the client as one choice in tackling an urge related presentation. If this approach was chosen, the client would be guided to identify a hierarchy of urge provoking situations related to her gambling then grade each situation in order of their intensity from low to high. After an brief period of stimulus control, she would expose herself to the least intense situation until she achieved mastery of her urge to succumb to the situation before moving to the next and so on. Graded Exposure could be offered to her through face-to-face sessions (Tolchard and Battersby 2013), via internet based programs (Rodda et al. 2013) or guided self-help printed media (Blaszczynski 2010). A second approach was explained to the client whereby she would work on stress reduction techniques that could help diminish the occurrences of her urge. However, it was explained that stress reduction alone would be unlikely to eradicate her urges entirely.

The client and worker together determined that the stress reduction techniques would be used as a secondary approach to the Graded Exposure to help prevent the urges from returning. Stress reduction techniques related to gambling triggers, as well as those focusing on general life stressors, are available (see—Helpguide.org). A Graded Exposure guide such as that offered by Blaszczynski (2010) was used.

#### Guiding

Guiding for this the client involved identification of her preferred approach to treatment and method for coaching. This client used a combination of face-to-face sessions (3–20 min treatment sessions) while she worked to formulate her selected approach for Graded Exposure, followed by six sessions of 10 min average length of telephone follow ups where the worker reviewed her progress, problem statement, intensity score, and goals. She worked through the hierarchal steps from low level urge producing situations of pictures of gambling, to sounds, imaginal encounters of being in gambling venues, actual encounters of being in the parking lot of a gambling venue with money in her pocket but not going into the venue, and going in the venue with money but not gambling. Over the first three face-to-face sessions, the intensity of her urge to gamble reduced from eight to five and she selected to have follow ups via phone. The coach offered encouragement on successes and suggestions for further homework. After the six telephone sessions, the client had achieved her goals and long-term follow-ups sessions at one, three, six and twelve months were planned.

### Case two: male, horse and EGM gambler

This case analysis involves a 46-year-old, single, and employed man living at home with his parents. At the time of the assessment his parents were about to ask him to leave the house.

#### Gathering

In the gathering step of this approach, the client was asked to describe his gambling problem using the five w’s and a ‘hot cross bun’ analysis was conducted. He described his experience as follows:

##### Five w’s

*What*. I have an excessive urge to gamble on the TAB (horse betting) and EGMs. *Where* I play the machines and bet on horses, in any venue with a TAB outlet. *When* I gamble any time I can, but mainly in the evenings whether I am working or not, as soon as the venue opens. *With whom* I always gamble alone. *Why* He describes his main triggers for gambling as “when I have money, when I am bored, or when I am reminded by passing a venue, by advertising, and by others talking about gambling”.

**Fig. 3:**
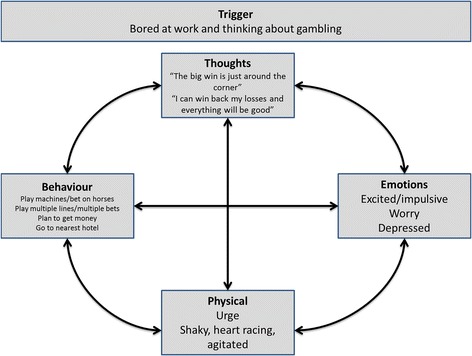
Five aspects of the ‘hot-cross bun’ for case 2

When he had money he had constant thoughts of gambling and how he could beat the system if he played in a particular way, leading to an urge to gamble. He described planning his next session, usually at the off track betting in a pub, so he could bet on horses and play the slot machines. He described feeling agitated, shaky, his heart racing, and an overwhelming feeling (urge) to gamble. When this occurred he went to the nearest hotel. With horse betting he studied the form guide and spread his bets across several horses based on a “system” he believed he has invented. On the slot machines he played multi-line or multi-bets preferring to continually play the same machine as this “increases my chances of winning”. When playing, he solely focused on the machines and played without stopping until all of his money has run out. Once he had lost his money he tried to get more in order to continue playing. “I always believe the big win is just around the corner”. When he began to lose he worried “how am I going to cope until I can get more money”. This lead him to feeling depressed (see Fig. 3).

*Impact* He experienced serious financial problems caused from borrowing from family and friends, leading to strained relationships. He had few belongings and lost his job, both as a result of the gambling. Further exacerbating his problems, he was now unable to find work, as many employers are aware of why he lost his previous job. In his gambling history he had experienced a single day win $11,000, and a single day loss of $1000. Overall, his total losses were $200,000 with a debt of $10,000 at the time of seeking treatment. When he was employed, he would frequently spend his entire salary in one session. *Suicidal intent* He had thoughts of suicide in the past which caused him to phone a Crisis Line. He threatened suicide following an argument with his sister. The method he threatened to use was cutting his wrists with a knife. This led to a siege with the police until he gave himself up. He was taken to a local mental health facility but was not admitted. Since this incident, and at the time of treatment, he had not had any suicidal ideation. *Onset* The gambling started ten years prior to seeking treatment, originally on TAB and Casino games and moving to EGMs once they were introduced. The move to playing EGMs resulted in his gambling becoming more serious. *Measures* He scored in the moderate range on all measures except the specific gambling screen (PHQ-9 = 18; GAD-7 = 10; WSAS = 3.5; Victorian Gambling Screen (VGS) = 25 (problem gambler)).

#### Generating

*Problem statement* I have thoughts of gambling constantly on my mind and whenever I have money my urge increases, so I plan how I can go to the local pub to place bets on horses and play the EGMs. I do so alone in the evenings and use my various systems to try and win the “big one” (8/8)

*Goal 1* To not respond to my thoughts of gambling by attending local pubs where I play TAB/EGMs (8/8)

*Goal 2* While carrying money, to be able to go out socially without responding to any thoughts of gambling (8/8)

#### Giving

This client was predominantly gambling due to the way he thought about the prospects of winning. While these thoughts also led to a physiological urge, the thoughts were the primary source of concern to him. Based on a range of cognitive materials offered and explained to him, the client selected to use the “Overcoming Your Pathological Gambling Workbook”—a Cognitive Restructuring (CR) manual for gambling (Ladouceur and Lachance 2007b). The workbook also has a companion guide for the worker or coach to use entitled “Overcoming Pathological Gambling—Therapist Guide” (Ladouceur and Lachance 2007a). Together the Workbook and Guide directed the client and worker to challenge beliefs of winning and how the client felt he could influence the outcome through his various systems. The main focus of the manual was to help him recognise the unhelpful impact that his thoughts were having on the problem.

#### Guiding

The worker coached the client in his use of the aforementioned manual over a period of 12 sessions—30 min per week face-to-face for 6 weeks followed by 6 sessions of phone coaching of 15-min length. This was longer than would be considered acceptable under a self-help model and did reflect the complexity of this client’s presentation. The 4G model while aiming for the least amount of contact is also designed to be individualistic and needs driven. While this client used a CR manual for gamblers, it would have been possible to adapt any general CR manual to challenge gamblers’ beliefs (see—Get.gg). The first task was to identify the nature of this gambler’s specific erroneous beliefs by first keeping a diary of the nature of, and times when, such thinking occurred. Once identified, the worker began to challenge the evidence to support the accuracy of his beliefs. The beliefs were tested through behavioural experiments whereby the client entered gambling venues and applied the new knowledge learned from the CR manual. Initially this client believed his specific gaming system could influence positive outcomes of a win, despite the evidence that the games were purely random, in particular with electronic game machines. The worker coached the client through the CR manual explanation of randomness.

## Conclusion

This paper presents a pragmatic framework to help workers from a range of health setting to access self-help materials for gamblers. The internet revolution has enabled a range of evidence-based health help to become widely available. The science around the specific cognitive, emotional and behavioral components of problem gambling and their requisite interventions for change are known (Cowlishaw et al. 2014). This article has provided a framework based on actual cases in which to help conceptualize a gambling problem and organize those self-help interventions using the 4G Framework. While not intended to provide definitive advice, it is hoped that a sufficient background on the common dynamics of problem gambling will help point workers and gamblers alike to explore the rich resources available to help to devise an effective self-help plan.

One of the main strengths of the New England 4G framework is that the individual makes the final decision on the approach that they will take, selecting from a range of possibilities that the worker has offered them (e.g., Cognitive Restructuring to change their thoughts about gambling, Behavioural Activation to increase pleasurable activity, or Graded Exposure to help reduce the urge to gamble when exposed to risk). This means that the individual has more of a sense of control over the process and is, therefore, more likely to be motivated to do the necessary work and make the necessary changes to their lives. Due to the explosion of self-help materials that are available on the internet, many of these can be incorporated into this framework. In this section, we will discuss the use of self-help treatment to help individuals to deal with their urge to gamble, their thoughts about gambling, their emotional responses to gambling, and their specific gambling behaviours. Finally, secondary issues related to gambling and their treatments will be discussed.

In order to deal with the urge to gamble that most individuals feel, the worker will need to identify possible urge reduction self-help programs, such as meditation materials relating to mindfulness. A recent review revealed that dispositional mindfulness has been shown to be of some benefit in reducing problem gambling (de Lisle et al. 2012). Mindfulness self-help materials exist (see-Get.gg), with some specifically designed for gambling (Cormier 2012).

Additionally, a number of websites exist that encourage gamblers to control their urge without using mindfulness. Gambling Help Online, the national help-line for problem gamblers in Australia, offers a range of advice including being able to call them 24 h a day (see—HelpGuide.org). The site follows the ‘Quit Smoking’ model, focusing on practical strategies to delay any response the individual may have to give into their urge (e.g., Tools for quitting). Similar sites are also available in the North America (e.g., Self Help Gambling Tools), United Kingdom (e.g., Gambling Therapy), New Zealand (e.g., Gambling Helpline) and parts of Europe (e.g., Gambling Helpline).

Self-exclusion is a gambling industry self-help approach which could be considered to manage the urge. This requires the gambler to complete a form, either face–face or online, which legally prevents them from returning to a particular gambling venue. This has been shown to be effective in initial urge reduction as a stimulus control process (Hing et al. 2014).

If the individual prefers solely to deal with their thoughts about gambling, then self-help based on Cognitive Restructuring is an appropriate option (Fortune and Goodie 2012). Many CR materials are available online and as self-help books. However, gambling specific materials should be provided to the client (Ladouceur and Lachance 2007a; Toneatto et al. 2003). Another useful cognitive approach is distraction which aims to remove the attention being given to gambling onto some other thought or activity. While this may be immediately useful, the ability to remain distracted is difficult. Therefore, as with self-exclusion, distraction may best be used as a stimulus control rather than a main line treatment (Oakes et al. 2012).

To help the person tackle their emotional responses, the worker will identify emotionally focussed resources and provide targeted help. In terms of controlling excitement and impulsivity, the worker can offer solutions including self-regulation, distraction techniques, mindfulness, and problem focussed coping (Chen et al. 2012). For the emotional response of shame, methods based on Acceptance and Commitment Therapy can be used (Hastings 2009). Using strategies that change the person’s response from an avoidant coping to an attribution style associated with guilt may also help alter the shame felt by gamblers (Yi and Kanetkar 2011).

When working on specific gambling behaviors, the worker can draw on a number of self-help materials. As discussed above, Graded Exposure encourages the person to control their behavior by directly challenging their desires in real life gambling venues, whereas Alternative Behavior Setting (Ladouceur et al. 1998; Petry et al. 2006) actively encourages gamblers to avoid such interaction. Both have their merits, although the first has a greater level of evidence supporting efficacy (Gooding and Tarrier 2009; Pallesen et al. 2005). Both approaches come in self-help materials and are readily available online or in print form (Ladouceur and Lachance 2007b; Lipinski et al. 2007). There may also be a benefit in encouraging the gambler to consider financial and relationship approaches (Brackertz 2014), using free online and paper based materials (see—GamblingHelpSA).

A secondary consequence of a person’s gambling may include reduced social interaction, low mood, lack of motivation and sleep and appetite disturbance. A number of non-gambling specific materials are available that can help the individual overcome such issues, including many well established techniques (e.g., Psychology Tools). Further, there are specific medical treatments the individual may be willing to pursue especially medications (Achab and Khazaal 2011). The most effective treatment is the use of Selective-Serotonin Reuptake Inhibitor anti-depressants (Saiz-Ruiz et al. 2005). There is clear evidence indicating their efficacy in moderating the gambler’s low mood, which it is argued makes it more likely for the individual to tackle their gambling problem more effectively (Pallesen et al. 2007). The use of Naltrexone, as with alcohol addiction, has shown some benefits and Lithium/anti-convulsion medications have helped reduce the impulsivity shown by gamblers (Pallanti et al. 2005). However, a major downside of any medication management includes side-effects, cost and lack of compliance (Choudhry and Shrank 2010; Julius et al. 2009).

In the final step of this approach, it is recognized that simply providing self-help materials alone is not enough. People maintain improvement when they continue to have some level of support from the worker (Rodda and Lubman 2012; Tse et al. 2013). In a number of countries there are national or state telephone help-lines and online help (e.g. Gambling Help Online (Australia), Gambling Therapy (United Kingdom) and physical support groups (e.g. Gamblers Anonymous and POKIES anonymous). These can be used for follow up sessions. However, we have found that a coaching session or a quick telephone check-in, to see how things are going, works well.
